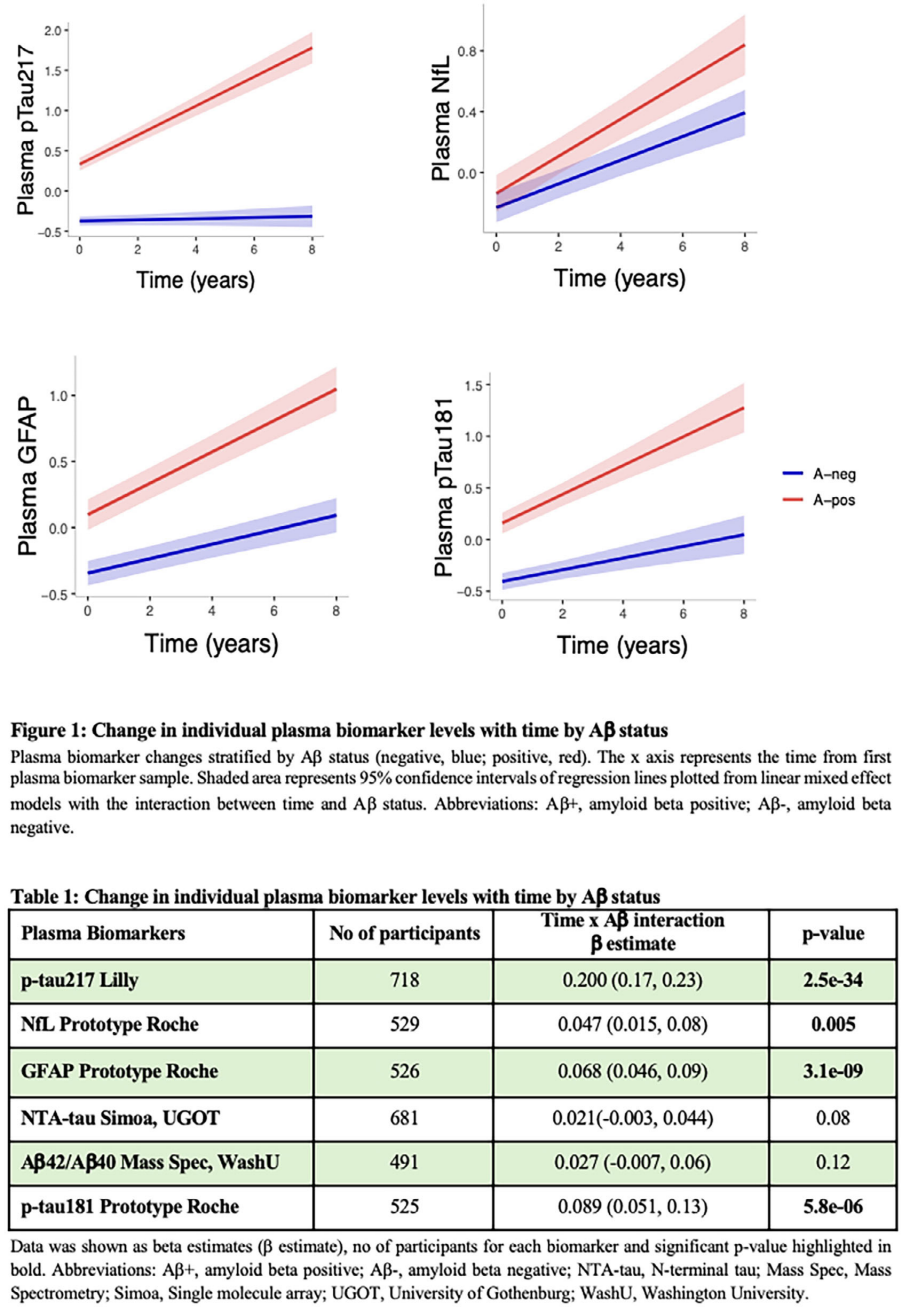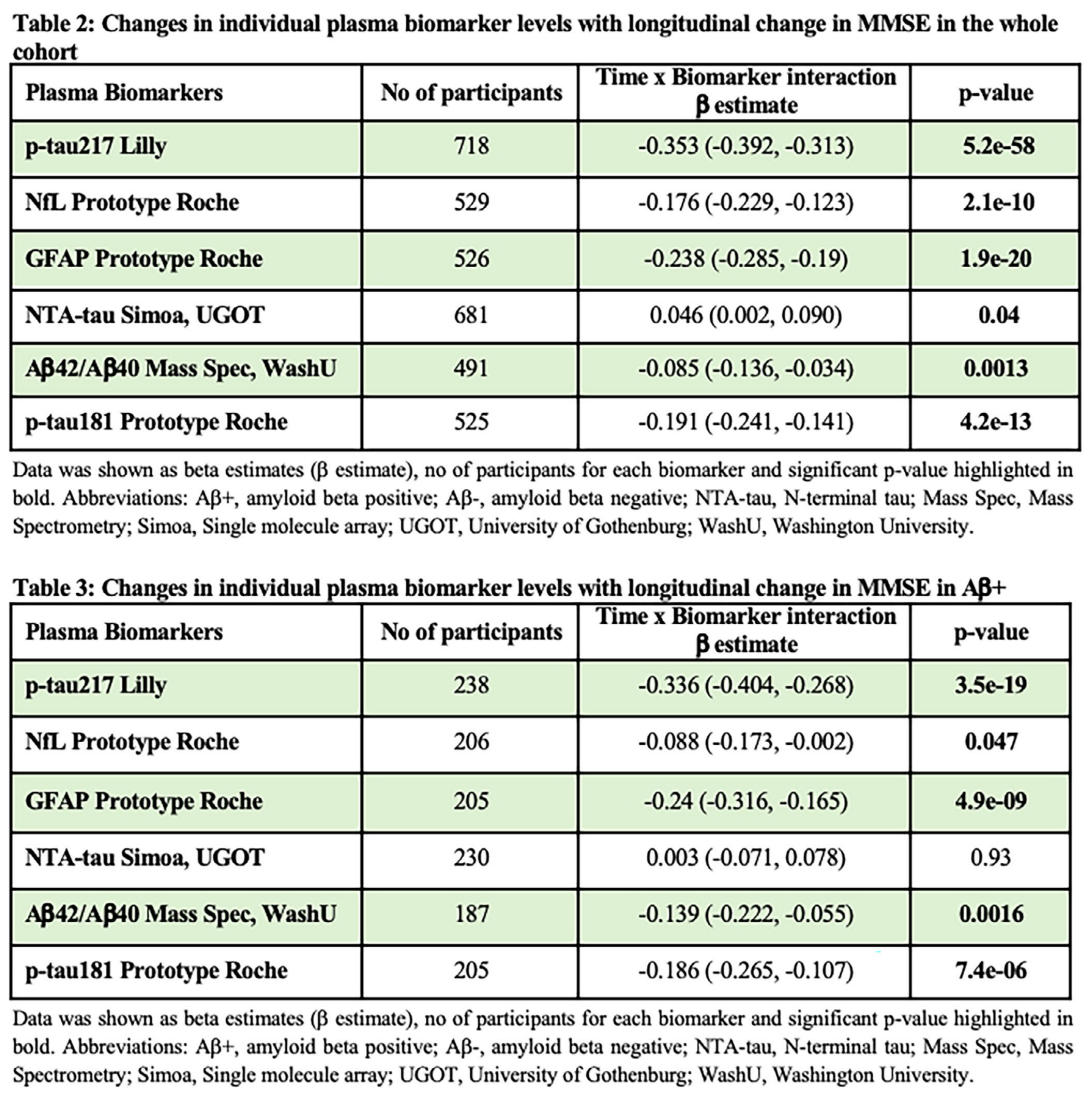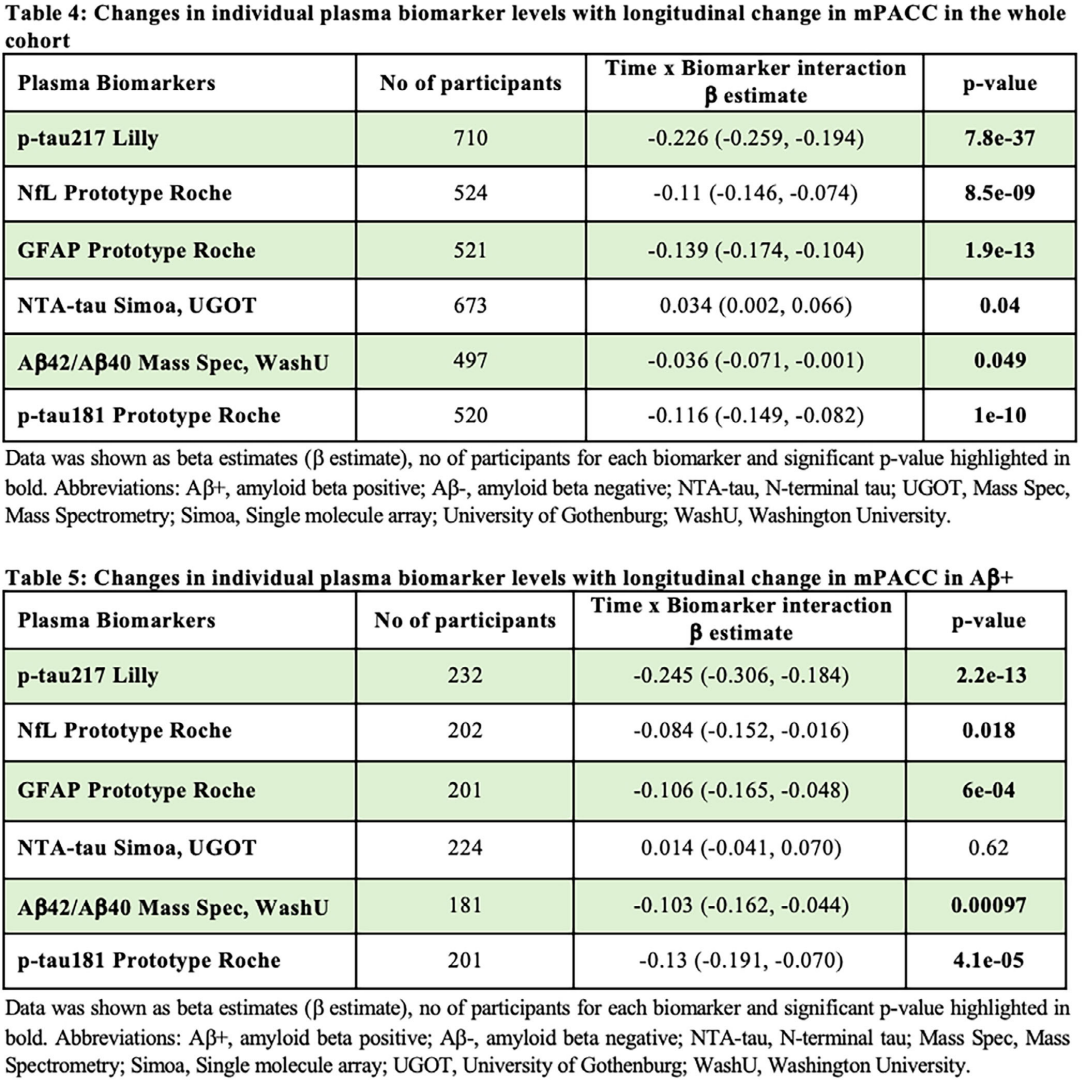# Can longitudinal measures of plasma biomarkers track disease progression in early Alzheimer´s Disease?

**DOI:** 10.1002/alz70856_105473

**Published:** 2026-01-08

**Authors:** Divya Bali, Shorena Janelidze, Gemma Salvadó, Nicholas J. Ashton, Sebastian Palmqvist, Juan Lantero Rodriguez, Erik Stomrud, Niklas Mattsson‐Carlgren, Oskar Hansson

**Affiliations:** ^1^ Clinical Memory Research Unit, Department of Clinical Sciences, Lund University, Lund, Skåne, Sweden; ^2^ Clinical Memory Research Unit, Department of Clinical Sciences Malmö, Faculty of Medicine, Lund University, Sweden, Lund, Sweden; ^3^ Clinical Memory Research Unit, Department of Clinical Sciences, Lund University, Lund, Sweden; ^4^ Barcelonaβeta Brain Research Center (BBRC), Pasqual Maragall Foundation, Barcelona, Spain; ^5^ Banner Sun Health Research Institute, Sun City, AZ, USA; ^6^ Memory Clinic, Skåne University Hospital, Malmö, Skåne, Sweden; ^7^ Clinical Memory Research Unit, Department of Clinical Sciences Malmö, Lund University, Lund, Sweden; ^8^ Department of Psychiatry and Neurochemistry, Institute of Neuroscience & Physiology, the Sahlgrenska Academy at the University of Gothenburg, Mölndal, Sweden; ^9^ Clinical Memory Research Unit, Lund University, Malmö, Skåne, Sweden; ^10^ Wallenberg Center for Molecular Medicine, Lund University, Lund, Sweden

## Abstract

**Background:**

Blood based biomarkers of Alzheimer´s disease (AD) that exhibit a high degree of change over time and are associated with deterioration in cognitive performance and atrophy could be useful in clinical trials to monitor treatment responses. In this study we investigated the longitudinal changes in plasma *p*‐tau biomarkers, amyloid‐beta(Aβ)42/Aβ40, Glial fibrillary acidic protein (GFAP) and Neurofilament light (NfL) and assessed associations between changes in these biomarkers and cognitive decline.

**Method:**

We included 718 participants (cognitively unimpaired (CU) or Mild Cognitive Impairment (MCI)) from the Swedish BioFINDER‐1 cohort who were followed upto 8 years. Plasma samples were analyzed for *p*‐tau217 (Lilly immunoassay), NfL, GFAP, *p*‐tau181 (Roche Prototype immunoassay), N‐terminal tau (NTA‐tau;Simoa immunoassay) and Aβ42/Aβ40 (mass spectrometry). We analyzed changes in individual plasma biomarker levels using linear mixed‐effects models, incorporating Aβ status*time interaction. Associations between biomarker slopes and cognitive decline (assessed with mini‐mental state examination [MMSE] and modified Preclinical Alzheimer´s Cognitive Composite[mPACC]) were assessed. An optimal model combining several biomarker slopes was also evaluated.

**Result:**

In the whole cohort, *p*‐tau217, NfL, GFAP and *p*‐tau181 were significantly increased over time showing more accelerated increase in Aβ+ than Aβ‐ participants. The strongest effect was seen for *p*‐tau217 (β=0.200, 95%CI0.17‐0.23;p<0.001) followed by NfL(β=0.047, 95%CI0.015‐0.08;p=0.005), GFAP(β=0.068, 95%CI0.046‐0.09; *p* = <0.001) and *p*‐tau181(β=0.089, 95%CI0.051,0.13;p<0.001)(Table 1, Figure 1). In individual models, slopes of all biomarkers were associated with change in MMSE and mPACC in the whole cohort. In the Aβ+ group, all biomarkers except NTA‐tau demonstrated significant associations with longitudinal MMSE and mPACC. The strongest associations were seen for *p*‐tau217 in both the whole cohort [MMSE (β=‐0.353, 95%CI ‐0.40‐(‐0.31)]; for interaction between *p*‐tau217 slope and time to predict cognitive scores; mPACC(β=‐0.226 95%CI‐0.26‐(‐0.19);p<0.001) and in the Aβ+ group [MMSE(β=‐0.336, 95%CI‐0.40‐(‐0.27); mPACC(β=‐0.245, 95%CI‐0.31‐(‐0.18);p<0.001)] (Tables 2‐5). The best performing model for predicting longitudinal MMSE and mPACC included the interaction between *p*‐tau217 slope (but not other biomarker slopes) and time, both in the whole cohort [MMSE(β=‐0.340,95%CI ‐0.39‐(‐0.30)); mPACC(β=‐0.238, 95%CI‐0.27‐(‐0.20);p<0.001)] and in the Aβ+ group [MMSE(β=‐0.327, 95%CI ‐0.40‐(‐0.25)]; mPACC(β=‐0.263, 95%CI ‐0.32‐(‐0.20); *p* <0.001)].

**Conclusion:**

The longitudinal changes in plasma *p*‐tau217 might serve as a surrogate marker for monitoring some aspects of disease progression during treatment trials.